# The Extended Erlang-Truncated Exponential distribution: Properties and application to rainfall data

**DOI:** 10.1016/j.heliyon.2017.e00296

**Published:** 2017-06-22

**Authors:** I.E. Okorie, A.C. Akpanta, J. Ohakwe, D.C. Chikezie

**Affiliations:** a School of Mathematics, University of Manchester, Manchester M13 9PL, UK; b Department of Statistics, Abia State University, Uturu, Abia State, Nigeria; c Department of Mathematics & Statistics, Faculty of Sciences, Federal University Otuoke, P.M.B 126 Yenagoa, Bayelsa, Nigeria

**Keywords:** Mathematics, Applied mathematics

## Abstract

The Erlang-Truncated Exponential **ETE** distribution is modified and the new lifetime distribution is called the Extended Erlang-Truncated Exponential **EETE** distribution. Some statistical and reliability properties of the new distribution are given and the method of maximum likelihood estimate was proposed for estimating the model parameters. The usefulness and flexibility of the **EETE** distribution was illustrated with an uncensored data set and its fit was compared with that of the **ETE** and three other three-parameter distributions. Results based on the minimized log-likelihood ($-\overset{ˆ}{\ell }$), Akaike information criterion (AIC), Bayesian information criterion (BIC) and the generalized Cramér–von Mises $W^{⋆}$ statistics shows that the **EETE** distribution provides a more reasonable fit than the one based on the other competing distributions.

## 1 Introduction

Erlang-Truncated Exponential (**ETE**) distribution was originally introduced by El-Alosey [1] as an extension of the standard one parameter exponential distribution. The **ETE** distribution results from the mixture of Erlang distribution and the left truncated one-parameter exponential distribution. The cumulative distribution function *(cdf) G(x)*, and probability density function *(pdf) g(x)* of the **ETE** distribution are given by;

(1) $$G(x)=1-e^{-\beta (1-e^{-\lambda })x};\;0\leq x<\infty ,\;\beta ,\;\lambda >0,$$

and

(2) $$g(x)=\beta (1-e^{-\lambda })e^{-\beta (1-e^{-\lambda })x};\;0\leq x<\infty ,\;\beta ,\;\lambda >0,$$

respectively, where *β* is the shape parameter and *λ* is the scale parameter. The **ETE** distribution collapses to the classical one-parameter exponential distribution with parameter *β* when λ→∞.

Unfortunately, the **ETE** distribution share the same limitation of constant failure rate property with the exponential distribution which makes it unsuitable for modelling many complex lifetime data sets that have nonconstant failure rate characteristics. Generally speaking, research has shown that the standard probability distributions are largely inadequate for modelling complex lifetime data sets and various excellent ways of overcoming this shortcoming have been proposed in the literature; for instance: Beta exponential G distributions, due to Alzaatreh et al. [2]; Beta extended G distributions, due to Cordeiro et al. [3]; Beta G distributions, due to Eugene et al. [4]; Exponentiated exponential Poisson G distributions, due to Ristić and Nadarajah [5]; Exponentiated generalized G distributions, due to Cordeiro et al. [6]; Marshall–Olkin G distributions, due to Marshall and Olkin [7]; Transmuted family of distributions, due to Shaw and Buckley [8]; and so on.

Mainly, by introducing extra shape parameter(s) to standard distribution a robust and more flexible distribution is derived. For a comprehensive list of methods of generating new distributions readers are encouraged to see Nadarajah and Rocha [9], AL-Hussaini, Ahsanullah [10], Ali et al. [11], Cordeiro et al. [12], Alzaatreh et al. [13] and Pescim et al. [14].

To motivate our new distribution, we consider the time to failure of a component in series arrangement of a certain device, denoted by *X* where $X_{1},X_{2},\cdots ,X_{\alpha }$ are independent and identically distributed **ETE** random variables. The device fails (stops functioning) if one of its component fails. Hence, the probability that the device will stop functioning before or exactly at a specified time say *x* is given by;

(3) $$\begin{array}{ccc} \text{P}(\max(X_{1},X_{2},\cdots ,X_{\alpha })\leq x) & = & \prod_{i=1}^{\alpha }\text{P}(X_{i}\leq x)\\ & = & \prod_{i=1}^{\alpha }F_{X_{i}}(x)\\ & = & {\left(F_{X_{i}}(x)\right)}^{\alpha }, \end{array}$$

this formulation fits exactly into the framework of Gupta and Kundu [15] (Exponentiated family of distributions).

The new distribution is called the Extended Erlang-Truncated Exponential (EETE) distribution. The **EETE** distribution has a tractable *pdf* whose shape is either decreasing or unimodal. The failure rate function (*frf*) is characterized by decreasing, constant and increasing shapes and the new three-parameter distribution demonstrates a superior fit when compared with some other well-known three-parameter distributions, as we shall see later. Related works are: the Transmuted Erlang-Truncated Exponential distribution, due to Okorie et al. [16], Marshall–Olkin generalized Erlang-truncated exponential distribution, due to Okorie et al. [17] and the generalized Erlang-Truncated Exponential distribution, due to Nasiru et al. [18].

The remaining part of this paper is organized as follows. In Section 2, we present the closed form mathematical expression for the *pdf* and *cdf* of the new probability distribution **EETE** and its statistical and reliability properties. In Section 3, the parameters of the **EETE** distribution are estimated through the method of maximum likelihood estimation. In Section 4, we perform a Monte-Carlo simulation study to assess the stability of the maximum likelihood estimates of the parameters of the **EETE** distribution. And we introduce a real data set, methods of model selection, application of the **EETE** distribution to the data and the results are also presented. In Section 5, we present the discussion of results, and lastly, in Section 6, we give the concluding remarks.

## 2 Model

The *cdf F(x)* and *pdf f(x)* of the **EETE** distribution are given by;

(4) $$F(x)={\left(1-e^{-\beta (1-e^{-\lambda })x}\right)}^{\alpha };\;0\leq x<\infty ,\;\alpha ,\;\beta ,\;\lambda >0,$$

and

(5) $$f(x)=\alpha \beta (1-e^{-\lambda })e^{-\beta (1-e^{-\lambda })x}{\left(1-e^{-\beta (1-e^{-\lambda })x}\right)}^{\alpha -1};\;0\leq x<\infty ,\;\alpha ,\;\beta ,\;\lambda >0,$$

where *α* and *β* are the shape parameters and *λ* is the scale parameter.

**Figure 1:**
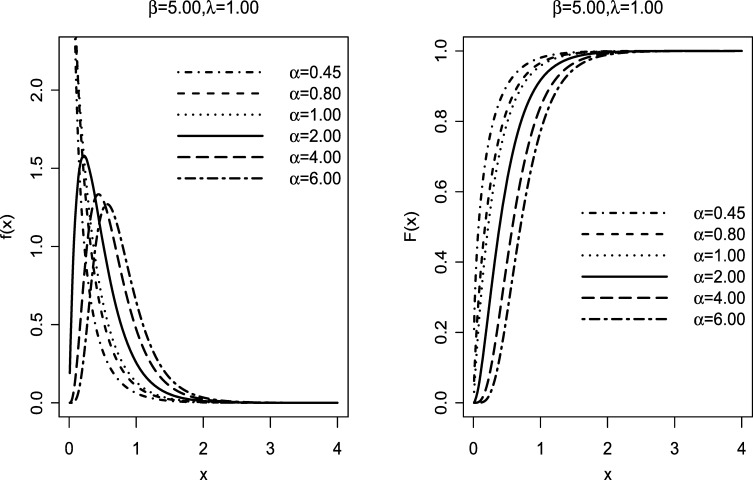
Possible shapes of the probability density function *f*(*x*) (*left*) and cumulative distribution function *F*(*x*) (*right*) of the **EETE** distribution for fixed parameter values of *β* and *λ*.

The **EETE** distribution reduces to the **ETE** distribution when α=1. The plots of the *cdf* and *pdf* are shown in Figure 1.

### 2.1 Reliability characteristics

The reliability function R(x) is an important tool for characterizing life phenomenon. R(x) is analytically expressed as R(x)=1−F(x). Under certain predefined conditions, the reliability function R(x) gives the probability that a system will operate without failure until a specified time *x*. The reliability function of the **EETE** distribution is given by;

(6) $$R(x)=1-{\left(1-e^{-\beta (1-e^{-\lambda })x}\right)}^{\alpha };\;0\leq x<\infty ,\;\alpha ,\;\beta ,\;\lambda >0.$$

Another important reliability characteristics is the failure rate function. The *frf* gives the probability of failure for a system that has survived up to time *x*. The *frf h(x)* is mathematically expressed as h(x)=f(x)/R(x). The *frf* of the **EETE** distribution is given by;

(7) $$h(x)=\frac{\alpha \beta (1-e^{-\lambda })e^{-\beta (1-e^{-\lambda })x}{\left(1-e^{-\beta (1-e^{-\lambda })x}\right)}^{\alpha -1}}{1-{\left(1-e^{-\beta (1-e^{-\lambda })x}\right)}^{\alpha }};\;0\leq x<\infty ,\;\alpha ,\;\beta ,\;\lambda >0.$$

**Figure 2:**
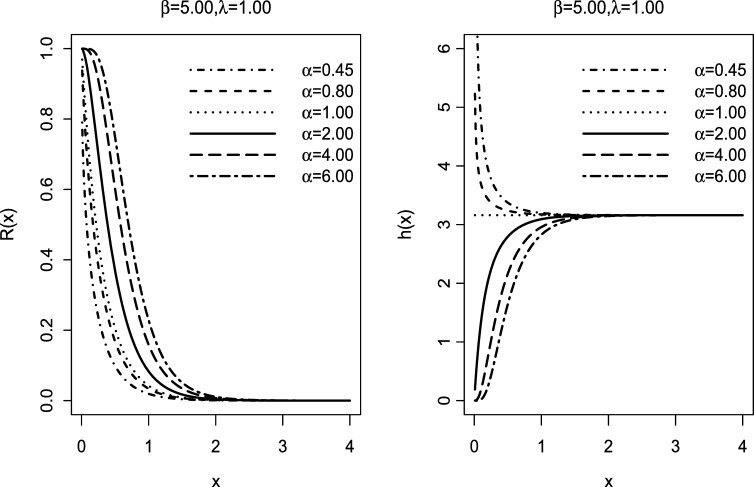
Possible shapes of the reliability function *R*(*x*) (*left*) and failure rate function *h*(*x*) (*right*) of the **EETE** distribution for fixed parameter values of *β* and *λ*.

The plots of the reliability function and *frf* are shown in Figure 2.

### 2.2 Asymptotics and shapes

In this section we present the asymptotics and shape characteristics of the *pdf* and *frf* of the **EETE** distribution. The following asymptotic behaviors are observed

$$f(0)=h(0)=h(\infty )=\left\{ \begin{array}{cc} \infty , & \text{if }\alpha <1,\\ \beta [1-e^{-\lambda }], & \text{if }\alpha =1;\text{ and }f(\infty )=0;\;\forall \;\alpha >0.\\ 0, & \text{if }\alpha >1, \end{array}\right.$$

Theorem 2.1 *A random variable X with pdf f(x) is said to be unimodal if the pdf is log-concave, i.e.;* $\log f^{\prime \prime }(x)\leq 0$*. If X* ∼ **EETE** *(*α,β,λ*) with pdf in Equation* (5) *then the distribution could take either of the two shapes*

$$\lim_{x\to \infty }f(x)=\left\{ \begin{array}{cc} \mathrm{decreasing}, & \mathrm{if}\;\alpha \leq 1,\\ \mathrm{unimodal}, & \mathrm{if}\;\alpha >1. \end{array}\right.$$

Proof Taking the log of the pdf in Equation (5) and differentiating *w.r.t x* gives

(8) $$\begin{array}{ccc} \log f(x) & = & \log(\alpha )+\log(\beta [1-e^{-\lambda }])-\beta [1-e^{-\lambda }]x+(\alpha -1)\log(1-e^{-\beta [1-e^{-\lambda }]x})\\ \log f^{\prime }(x) & = & -\beta [1-e^{-\lambda }]+\frac{(\alpha -1)\beta [1-e^{-\lambda }]e^{-\beta [1-e^{-\lambda }]x}}{1-e^{-\beta [1-e^{-\lambda }]x}}. \end{array}$$

From Equation (8) it is clear that the *pdf* of the **EETE** distribution is decreasing as x→∞ when α<1 and $f^{\prime }(x)$ has a single root, denoted by $x_{0}$ when α>1 and the root is located at

(9) $$x_{0}=\frac{\log(\alpha )}{2\beta [1-e^{-\lambda }]};\;\alpha >1,$$

this implies that there exist some $x<x_{0}$ such that f(x) is increasing and $x>x_{0}$ such that f(x) is decreasing, then $x_{0}$ is said to be the critical point at which the *pdf* is maximized (i.e.; the mode). The second derivative

$$\log f^{\prime \prime }(x)={\frac{-(\alpha -1){\left(\beta [1-e^{-\lambda }]\right)}^{2}e^{-\beta [1-e^{-\lambda }]x}}{{\left(1-e^{-\beta [1-e^{-\lambda }]x}\right)}^{2}}<0|}_{\alpha >1},$$

completes the proof. □

Theorem 2.2 *If X follows the* **EETE** *distribution with* frf *in Equation* (7) *then the shape of the* frf h(x) *are*

$$\lim_{x\to \infty }h(x)=\left\{ \begin{array}{cc} \mathrm{constant}(\beta [1-e^{-\lambda }]), & \mathrm{if}\;\alpha =1,\\ \mathrm{decreasing}, & \mathrm{if}\;\alpha <1,\\ \mathrm{increasing}, & \mathrm{if}\;\alpha >1. \end{array}\right.$$

Proof The first case is satisfied when Equation (7) is evaluated at α=1. The first derivative of the log of h(x) is

(10) $$\begin{array}{ccc} \log h(x) & = & \log(\alpha )+\log(\beta )+\log(1-e^{-\lambda })-\beta [1-e^{-\lambda }]x\\ & & +(\alpha -1)\log(1-e^{-\beta [1-e^{-\lambda }]x})+\alpha \log(1-e^{-\beta [1-e^{-\lambda }]x})\\ \log h^{\prime }(x) & = & -\beta [1-e^{-\lambda }]+\frac{(\alpha -1)\beta [1-e^{-\lambda }]e^{-\beta [1-e^{-\lambda }]x}}{1-e^{-\beta [1-e^{-\lambda }]x}}\\ & & +\frac{\alpha \beta [1-e^{-\lambda }]e^{-\beta [1-e^{-\lambda }]x}}{1-e^{-\beta [1-e^{-\lambda }]x}}. \end{array}$$

It is clear from Equation (10) that the failure rate of the **EETE** distribution is decreasing when α<1 and increasing when α>1 and Equation (10) has no unique root. □

### 2.3 The *qth* quantile function

The *qth* quantile function of the **EETE** distribution is given by;

(11) $$x_{q}=\frac{-\log(1-q^{\frac{1}{\alpha }})}{\beta (1-e^{-\lambda })};\;q\in (0,1).$$

Random samples from the **EETE** distribution can be simulated through the inversion of *cdf* method by simply substituting *q* with a uniform U(0,1) variates. It is easy to obtain the median of the **EETE** distribution by substituting q=1/2 in Equation (11). For any given values of *α*, *β* and *λ*, the median of the **EETE** distribution is given by;

$$x_{0.5}=\frac{-\log(1-0.5^{\frac{1}{\alpha }})}{\beta (1-e^{-\lambda })}.$$

### 2.4 The *kth* crude moment

Theorem 2.3 *If the kth crude moment of any random variable X exists then other essential characteristics of the distribution could be calculated; for example the mean, variance, coefficient of variation, skewness and kurtosis statistics. The kth crude moment of a continuous random variable X is defined by* $E(X^{k})=\mu_{k}^{\prime }=\int_{-\infty }^{\infty }x^{k}f(x)dx$*. Hence, it follows that the kth crude moment of the* **EETE** *distribution is*

$$\mu_{k}^{\prime }=\frac{\alpha \alpha !k!}{{\left[\beta (1-e^{-\lambda })\right]}^{k}}\sum_{i,j=0}^{\infty }\frac{{\left(-1\right)}^{i+2j}}{i!(\alpha -i)!{\left(i+j+1\right)}^{k+1}}.$$

Proof Using Equation (5) and the definition of $\mu_{k}^{\prime }$ we have

(12) $$\begin{array}{ccc} \mu_{k}^{\prime } & = & \int_{0}^{\infty }x^{k}\alpha \beta (1-e^{-\lambda })e^{-\beta (1-e^{-\lambda })x}{\left(1-e^{-\beta (1-e^{-\lambda })x}\right)}^{\alpha -1}dx\\ & = & \alpha \beta (1-e^{-\lambda })\int_{0}^{\infty }x^{k}e^{-\beta (1-e^{-\lambda })x}{\left(1-e^{-\beta (1-e^{-\lambda })x}\right)}^{\alpha -1}dx, \end{array}$$

substituting $y=\beta (1-e^{-\lambda })x$ into Equation (12) we have

(13) $$\mu_{k}^{\prime }=\frac{\alpha }{{\left[\beta (1-e^{-\lambda })\right]}^{k}}\int_{0}^{\infty }y^{k}e^{-y}{\left(1-e^{-y}\right)}^{\alpha -1}dy,$$

the series expansion of ${\left(1-y\right)}^{r-1}$ for |y|<1 and $r\in R^{+}$(non-integer) could be expressed as

$$\begin{array}{ccc} {\left(1-y\right)}^{r-1} & = & {\left(1-y\right)}^{r}\cdot {\left(1-y\right)}^{-1}\\ & = & \sum_{i=0}^{\infty }\left(\begin{array}{c} r\\ i \end{array}\right){\left(-1\right)}^{i}y^{i}\cdot \sum_{j=0}^{\infty }{\left(-1\right)}^{j}\left(\begin{array}{c} 1+j-1\\ j \end{array}\right){\left(-1\right)}^{j}y^{j}\\ & = & \sum_{i=0}^{\infty }\sum_{j=0}^{\infty }{\left(-1\right)}^{i+2j}\left(\begin{array}{c} r\\ i \end{array}\right)y^{i+j}\\ & = & r!\sum_{i,j=0}^{\infty }{\left(-1\right)}^{i+2j}\frac{y^{i+j}}{i!(r-i)!}\\ & = & \Gamma (r+1)\sum_{i,j=0}^{\infty }{\left(-1\right)}^{i+2j}\frac{y^{i+j}}{\Gamma (i+1)\cdot \Gamma (r-i+1)}. \end{array}$$

If $(r>1)\in R^{+}$(integer) then the sum terminates at r−1; thus, the series representation of Equation (13) is given by;

(14) $$\begin{array}{ccc} \mu_{k}^{\prime } & = & \frac{\alpha }{{\left[\beta (1-e^{-\lambda })\right]}^{k}}\int_{0}^{\infty }y^{k}e^{-y}\sum_{i=0}^{\infty }\left(\begin{array}{c} \alpha \\ i \end{array}\right){\left(-1\right)}^{i}e^{-yi}\sum_{j=0}^{\infty }{\left(-1\right)}^{j}\left(\begin{array}{c} 1+j-1\\ j \end{array}\right){\left(-1\right)}^{j}e^{-yj}dy\\ & = & \frac{\alpha }{{\left[\beta (1-e^{-\lambda })\right]}^{k}}\sum_{i,j=0}^{\infty }{\left(-1\right)}^{i+2j}\left(\begin{array}{c} \alpha \\ i \end{array}\right)\int_{0}^{\infty }y^{k}e^{-y(i+j+1)}dy \end{array}$$

and by substituting z=y(i+j+1) into Equation (14) we have

(15) $$\begin{array}{ccc} \mu_{k}^{\prime } & = & \frac{\alpha }{{\left[\beta (1-e^{-\lambda })\right]}^{k}}\sum_{i,j=0}^{\infty }\frac{{\left(-1\right)}^{i+2j}}{{\left(i+j+1\right)}^{k+1}}\left(\begin{array}{c} \alpha \\ i \end{array}\right)\int_{0}^{\infty }z^{k}e^{-z}dz\\ & = & \frac{\alpha \alpha !k!}{{\left[\beta (1-e^{-\lambda })\right]}^{k}}\sum_{i,j=0}^{\infty }\frac{{\left(-1\right)}^{i+2j}}{i!(\alpha -i)!{\left(i+j+1\right)}^{k+1}}. \end{array}$$

 □

The mean is a very important characteristics of a distribution not only in statistics but also in reliability engineering as it is referred to as the mean time to failure (**MTTF**). Under some predefined conditions **MTTF** could be interpreted as the expected length of time a non-repairable item can operate before it fails. The mean of the **EETE** distribution is given by;

$$\mu_{1}^{\prime }=\frac{\alpha \alpha !}{\beta (1-e^{-\lambda })}\sum_{i,j=0}^{\infty }\frac{{\left(-1\right)}^{i+2j}}{i!(\alpha -i)!{\left(i+j+1\right)}^{2}}$$

and the variance V(X) could be obtained by

$$\begin{array}{ccc} V(X) & = & \frac{2\alpha \alpha !}{{\left[\beta (1-e^{-\lambda })\right]}^{2}}\sum_{i,j=0}^{\infty }\frac{{\left(-1\right)}^{i+2j}}{i!(\alpha -i)!{\left(i+j+1\right)}^{3}}\\ & & -{\left(\frac{\alpha \alpha !}{\beta (1-e^{-\lambda })}\sum_{i,j=0}^{\infty }\frac{{\left(-1\right)}^{i+2j}}{i!(\alpha -i)!{\left(i+j+1\right)}^{2}}\right)}^{2}. \end{array}$$

The coefficient of variation (CV), skewness $\left(\gamma_{1}\right)$ and kurtosis $\left(\gamma_{2}\right)$ statistics of the **EETE** distribution could be obtained by evaluating

$$\begin{array}{ccc} CV & = & \sqrt{\frac{\mu_{2}^{\prime }}{\mu_{1}^{\prime 2}}-1},\\ \gamma_{1} & = & \frac{\mu_{3}^{\prime }-3\mu_{2}^{\prime }\mu_{1}^{\prime }+2\mu_{1}^{\prime 3}}{{\left(\mu_{2}^{\prime }-\mu_{1}^{\prime 2}\right)}^{\frac{3}{2}}}, \end{array}$$

and

$$\gamma_{2}=\frac{\mu_{4}^{\prime }-4\mu_{3}^{\prime }\mu_{1}^{\prime }+6\mu_{2}^{\prime }\mu_{1}^{\prime 2}-3\mu_{1}^{\prime 4}}{{\left(\mu_{2}^{\prime }-\mu_{1}^{\prime 2}\right)}^{2}}$$

respectively.

Comparing the results in Table 1 with those in Table 2 we observe that ∀ *β* and *λ*, $\mu_{k\;\mathrm{EETE}}^{\prime }=\mu_{k\;\mathrm{ETE}}^{\prime }$ when α=1, $\mu_{k\;\mathrm{EETE}}^{\prime }<\mu_{k\;\mathrm{ETE}}^{\prime }$ for all α<1, and ∀ α>1 $\mu_{k\;\mathrm{EETE}}^{\prime }>\mu_{k\;\mathrm{ETE}}^{\prime }$.

**Table 1:** The first four order moments of 200 random samples from the **EETE** distribution with selected parameter values.

α,β,λ↓	$\mu_{1}^{\prime }$	$\mu_{2}^{\prime }$	$\mu_{3}^{\prime }$	$\mu_{4}^{\prime }$
3.5,5,0.2	2.1692	6.4053	24.4149	115.6766
0.01,2,3	0.0086	0.0066	0.0095	0.0191
0.45,5,10	0.1132	0.0395	0.0225	0.0176
0.001,0.5,20	0.0033	0.0096	0.0519	0.3982
3,12,8	0.1528	0.0328	0.0093	0.0033
0.5,0.5,0.5	3.1195	28.0785	408.9417	8151.614
28,7,1	0.8875	0.8699	0.9458	1.1448
0.8,1,50	0.8622	1.6514	4.8698	19.3318
4,0.45,.01	465.2816	287494.7	226080693	219160499642
1,5,5	0.2014	0.0811	0.049	0.0395

**Table 2:** The first four order moments of 200 random samples from the **ETE** distribution with selected parameter values.

β,λ↓	$\mu_{1}^{\prime }$	$\mu_{2}^{\prime }$	$\mu_{3}^{\prime }$	$\mu_{4}^{\prime }$
5,0.2	1.1033	2.4347	8.0588	35.566
2,3	0.5262	0.5538	0.8742	1.84
5,10	0.2	0.08	0.048	0.0384
0.5,20	2	8	48	384
12,8	0.0834	0.0139	0.0035	0.0012
0.5,0.5	5.083	51.6735	787.9679	16020.93
7,1	0.226	0.1021	0.0693	0.0626
1,50	1	2	6	24
0.45,0.01	223.3352	99757.21	66837885	59709005539
5,5	0.2014	0.0811	0.049	0.0395

The following **R** code was used to produce the results in Table 1, and the results in Table 2 can be reproduced on slight modification of this code.

moments<-function(n,k,alpha,beta,lambda)  {  Q<-runif(n)  x<--log(1-Q**(1/alpha))/(beta*(1-exp(-lambda)))  x  f<-function(x,k,alpha,beta,lambda)  {  y<-x**k*alpha*dexp(x,beta*(1-exp(-lambda)))*  (pexp(x,beta*(1-exp(-lambda))))**(alpha-1)  y  }  z<-integrate(f,lower=0,upper=Inf,k=k,alpha=alpha,  beta=beta,lambda=lambda)$value  z  }

### 2.5 The moment generating function

Corollary 2.3.1 *The moment generating function is generally defined by*

(16) $$M_{X}(t)=E(e^{tx})=E\left(\sum_{k=0}^{\infty }\frac{{\left(tx\right)}^{k}}{k!}\right)=\sum_{k=0}^{\infty }\frac{t^{k}}{k!}\mu_{k}^{\prime }.$$

*It is clear that we can obtain the mgf of the* **EETE** *distribution by substituting Equation* (15) *into* (16) *as*

$$M_{X}(t)=\alpha \alpha !\sum_{i,j,k=0}^{\infty }\frac{{\left(-1\right)}^{i+2j}}{i!(\alpha -i)!{\left(i+j+1\right)}^{k+1}}{\left[\frac{t}{\beta (1-e^{-\lambda })}\right]}^{k}.$$

### 2.6 The *rth* incomplete moment

Theorem 2.4 *If a random variable is distributed according to the* **EETE** *distribution then its rth incomplete moment denoted by* I(x;r) *is given by;*

$$I(X;r)={\frac{4\alpha }{{\left[\beta (1-e^{-\lambda })\right]}^{r}}\frac{\partial^{r}}{\partial \ell^{r}}e^{\pi i(\ell +\alpha +1)}\sin(\pi (\ell +1))\sin(\pi \alpha )B(\ell +1,\alpha )|}_{\ell =0},$$

*where* B(⋅,⋅) *is the Beta function.*

Proof Using Equation (5) and the definition of $I(x;r)=\int_{-\infty }^{x}x^{r}f(x)dx$ we have

(17) $$I(X;r)=\alpha \beta (1-e^{-\lambda })\int_{0}^{x}y^{r}e^{-\beta (1-e^{-\lambda })y}{\left(1-e^{-\beta (1-e^{-\lambda })y}\right)}^{\alpha -1}dy,$$

setting $z=\beta (1-e^{-\lambda })y$ into Equation (17) we have

(18) $$I(X;r)=\frac{\alpha }{{\left[\beta (1-e^{-\lambda })\right]}^{r}}\int_{0}^{\beta (1-e^{-\lambda })x}z^{r}e^{-z}{\left(1-e^{-z}\right)}^{\alpha -1}dz,$$

setting $u=e^{-z}$ into Equation (18) gives

$$\begin{array}{ccc} I(X;r) & = & \frac{\alpha }{{\left[\beta (1-e^{-\lambda })\right]}^{r}}\int_{1}^{e^{-\beta (1-e^{-\lambda })x}}\log{\left(u\right)}^{r}{\left(1-u\right)}^{\alpha -1}du\\ & = & -{\frac{\alpha }{{\left[\beta (1-e^{-\lambda })\right]}^{r}}\frac{\partial^{r}}{\partial \ell^{r}}\underset{\text{Pochhammer contour integral}}{\underset{︸}{\int_{e^{-\beta (1-e^{-\lambda })x}}^{1}u^{\ell }{\left(1-u\right)}^{\alpha -1}du}}|}_{\ell =0}\\ & = & {\frac{4\alpha }{{\left[\beta (1-e^{-\lambda })\right]}^{r}}\frac{\partial^{r}}{\partial \ell^{r}}e^{\pi i(\ell +\alpha +1)}\sin(\pi (\ell +1))\sin(\pi \alpha )B(\ell +1,\alpha )|}_{\ell =0} \end{array}$$

where the Pochhammer contour integral is defined by

$$\begin{array}{c} \int_{P}^{\left(1+,0+,1-,0-\right)}v^{\xi -1}{\left(1-v\right)}^{\omega -1}dv=-4e^{\pi i(\xi +\omega )}\sin(\pi \xi )\sin(\pi \omega )B(\xi ,\omega );\\ \;P\in (0,1),i=\sqrt{-1} \end{array}$$

in Equation (5.12.12) of Frank et al. [19]. □

### 2.7 The *kth* central moment

Theorem 2.5 *The kth central moment of a continuous random variable X is defined by* $E({\left(X-\mu \right)}^{k})=\int_{-\infty }^{\infty }{\left(X-\mu \right)}^{k}f(x)dx$*. Hence, the kth central moment of the* **EETE** *distribution is*

$$E{\left(X-\mu \right)}^{k}=\frac{\alpha \alpha !k!}{{\left[\beta (1-e^{-\lambda })\right]}^{k}}\sum_{\ell ,m,n=0}^{\infty }{\left(-1\right)}^{\ell +m+2n}\frac{{\left[\mu \beta (1-e^{-\lambda })\right]}^{\ell }}{{\left(m+n+1\right)}^{k-\ell +1}\ell !m!(\alpha -m)!}.$$

Proof Using Equation (5) and the definition of $E({\left(x-\mu \right)}^{k})$ we have

(19) $$\begin{array}{ccc} E{\left(X-\mu \right)}^{k} & = & \int_{0}^{\infty }{\left(x-\mu \right)}^{k}\alpha \beta (1-e^{-\lambda })e^{-\beta (1-e^{-\lambda })x}{\left(1-e^{-\beta (1-e^{-\lambda })}\right)}^{\alpha -1}dx\\ & = & \alpha \beta (1-e^{-\lambda })\int_{0}^{\infty }{\left(x-\mu \right)}^{k}e^{-\beta (1-e^{-\lambda })x}{\left(1-e^{-\beta (1-e^{-\lambda })}\right)}^{\alpha -1}dx, \end{array}$$

substituting $y=\beta (1-e^{-\lambda })x$ into Equation (19) we have

(20) $$E{\left(X-\mu \right)}^{k}=\alpha {\left[\beta (1-e^{-\lambda })\right]}^{-k}\int_{0}^{\infty }{\left(y-\mu \beta (1-e^{-\lambda })\right)}^{k}e^{-y}{\left(1-e^{-y}\right)}^{\alpha -1}dy,$$

the series expansion of Equation (20) is given by;

(21) $$\begin{array}{ccc} E{\left(X-\mu \right)}^{k} & = & \frac{\alpha }{{\left[\beta (1-e^{-\lambda })\right]}^{k}}\int_{0}^{\infty }e^{-y}\sum_{\ell =0}^{\infty }\left(\begin{array}{c} k\\ \ell \end{array}\right){\left(-1\right)}^{\ell }{\left[\mu \beta (1-e^{-\lambda })\right]}^{\ell }y^{k-\ell }\\ & & \times \sum_{m=0}^{\infty }\left(\begin{array}{c} \alpha \\ m \end{array}\right){\left(-1\right)}^{m}e^{-ym}\sum_{n=0}^{\infty }{\left(-1\right)}^{n}\left(\begin{array}{c} 1+n-1\\ n \end{array}\right){\left(-1\right)}^{n}e^{-yn}dy\\ & = & \frac{\alpha }{{\left[\beta (1-e^{-\lambda })\right]}^{k}}\sum_{\ell ,m,n=0}^{\infty }{\left(-1\right)}^{\ell +m+2n}\left(\begin{array}{c} k\\ \ell \end{array}\right) \end{array}$$

(22) $$\times \left(\begin{array}{c} \alpha \\ m \end{array}\right){\left[\mu \beta (1-e^{-\lambda })\right]}^{\ell }\int_{0}^{\infty }y^{k-\ell }e^{-y(m+n+1)}dy$$

and by substituting z=y(m+n+1) into Equation (22) we obtain

$$\begin{array}{ccc} E{\left(X-\mu \right)}^{k} & = & \frac{\alpha }{{\left[\beta (1-e^{-\lambda })\right]}^{k}}\\ & & \times \sum_{\ell ,m,n=0}^{\infty }{\left(-1\right)}^{\ell +m+2n}\left(\begin{array}{c} k\\ \ell \end{array}\right)\left(\begin{array}{c} \alpha \\ m \end{array}\right)\frac{{\left[\mu \beta (1-e^{-\lambda })\right]}^{\ell }}{{\left(m+n+1\right)}^{k-\ell +1}}\int_{0}^{\infty }z^{k-\ell }e^{-z}dz\\ & = & \frac{\alpha }{{\left[\beta (1-e^{-\lambda })\right]}^{k}}\\ & & \times \sum_{\ell ,m,n=0}^{\infty }{\left(-1\right)}^{\ell +m+2n}\left(\begin{array}{c} k\\ \ell \end{array}\right)\left(\begin{array}{c} \alpha \\ m \end{array}\right)\frac{{\left[\mu \beta (1-e^{-\lambda })\right]}^{\ell }}{{\left(m+n+1\right)}^{k-\ell +1}}\int_{0}^{\infty }z^{k-\ell }e^{-z}dz\\ & = & \frac{\alpha \alpha !k!}{{\left[\beta (1-e^{-\lambda })\right]}^{k}}\\ & & \times \sum_{\ell ,m,n=0}^{\infty }{\left(-1\right)}^{\ell +m+2n}\frac{{\left[\mu \beta (1-e^{-\lambda })\right]}^{\ell }\Gamma (k-\ell +1)}{{\left(m+n+1\right)}^{k-\ell +1}\ell !m!(k-\ell )!(\alpha -m)!} \end{array}$$

and finally

$$\begin{array}{ccc} E{\left(X-\mu \right)}^{k} & = & \frac{\alpha \alpha !k!}{{\left[\beta (1-e^{-\lambda })\right]}^{k}}\\ & & \times \sum_{\ell ,m,n=0}^{\infty }{\left(-1\right)}^{\ell +m+2n}\frac{{\left[\mu \beta (1-e^{-\lambda })\right]}^{\ell }}{{\left(m+n+1\right)}^{k-\ell +1}\ell !m!(\alpha -m)!}. \end{array}$$

 □

### 2.8 Rényi entropy measure

Theorem 2.6 *The Rényi entropy is used to quantify the uncertainty of variation in a random variable X and the Rényi entropy measure of a continuous random variable is generally given by;*

(23) $$I_{R}(\theta )=\frac{1}{1-\theta }\log\left(\;\int_{-\infty }^{\infty }f^{\theta }(x)dx\right)\;\mathrm{for}\;\theta >0\backslash \{1\}.$$

*Hence, if X is distributed according to the* **EETE** *distribution its Rényi entropy measure will be given by;*

$$I_{R}(\theta )=\frac{1}{1-\theta }\log\left[\alpha^{\theta }(\theta \alpha )!{\left[\beta (1-e^{-\lambda })\right]}^{\theta -1}\sum_{i,j=0}^{\infty }\frac{{\left(-1\right)}^{i+2j}(\theta +j-1)!}{(i+j+\theta )i!j!(\theta \alpha -i)!(\theta -1)!}\right].$$

Proof Using Equation (5) we first evaluate the integral part of Equation (23) as follows

(24) $$\begin{array}{ccc} \int_{0}^{\infty }f^{\theta }(x)dx & = & \int_{0}^{\infty }{\left[\alpha \beta (1-e^{-\lambda })e^{-\beta (1-e^{-\lambda })x}{\left(1-e^{-\beta (1-e^{-\lambda })x}\right)}^{\alpha -1}\right]}^{\theta }dx\\ & = & {\left[\alpha \beta (1-e^{-\lambda })\right]}^{\theta }\int_{0}^{\infty }e^{-\theta \beta (1-e^{-\lambda })x}{\left(1-e^{-\beta (1-e^{-\lambda })x}\right)}^{\theta \alpha -\theta }dx, \end{array}$$

substituting $y=\beta (1-e^{-\lambda })x$ into Equation (24) gives

(25) $$\int_{0}^{\infty }f^{\theta }(x)dx=\alpha^{\theta }{\left[\beta (1-e^{-\lambda })\right]}^{\theta -1}\int_{0}^{\infty }e^{-\theta y}{\left(1-e^{-y}\right)}^{\theta \alpha -\theta }dy$$

and the series expansion of Equation (25) is given by;

$$\begin{array}{ccc} \int_{0}^{\infty }f^{\theta }(x)dx & = & \alpha^{\theta }{\left[\beta (1-e^{-\lambda })\right]}^{\theta -1}\\ & & \times \int_{0}^{\infty }e^{-\theta y}\sum_{i=0}^{\infty }\left(\begin{array}{c} \theta \alpha \\ i \end{array}\right){\left(-1\right)}^{i}e^{-yi}\sum_{j=0}^{\infty }{\left(-1\right)}^{j}\left(\begin{array}{c} \theta +j-1\\ j \end{array}\right){\left(-1\right)}^{j}e^{-yj}dy\\ & = & \alpha^{\theta }{\left[\beta (1-e^{-\lambda })\right]}^{\theta -1}\\ & & \times \sum_{i,j=0}^{\infty }{\left(-1\right)}^{i+2j}\left(\begin{array}{c} \theta \alpha \\ i \end{array}\right)\left(\begin{array}{c} \theta +j-1\\ j \end{array}\right)\int_{0}^{\infty }e^{-y(i+j+\theta )}dy\\ & = & \alpha^{\theta }(\theta \alpha )!{\left[\beta (1-e^{-\lambda })\right]}^{\theta -1}\\ & & \times \sum_{i,j=0}^{\infty }{\left(-1\right)}^{i+2j}\frac{(\theta +j-1)!}{(i+j+\theta )i!j!(\theta \alpha -i)!(\theta -1)!} \end{array}$$

therefore; the Rényi entropy measure of the **EETE** distribution is given by;

$$\begin{array}{c} I_{R}(\theta )\\ =\frac{1}{1-\theta }\log\left[\alpha^{\theta }(\theta \alpha )!{\left[\beta (1-e^{-\lambda })\right]}^{\theta -1}\sum_{i,j=0}^{\infty }\frac{{\left(-1\right)}^{i+2j}(\theta +j-1)!}{(i+j+\theta )i!j!(\theta \alpha -i)!(\theta -1)!}\right]. \end{array}$$

 □

### 2.9 Order statistics

Order statistics is an essential tool in reliability and life testing analysis. Suppose the following *n*-sized random sample $X_{1},X_{2},\ldots ,X_{n}$ was drawn from the **EETE** distribution with *pdf* and *cdf* corresponding to Equation (5) and (4). Let $X_{1,n}<X_{2,n}<\ldots <X_{n,n}$ represent the *ith* order statistics denoted by $X_{i,n}$ then, $X_{i,n}$ could be interpreted as the lifetime of the (n−i+1)th item of the total *nth* independent and identical components. The density of $X_{i,n}$ could be expressed as

(26) $$f_{i,n}(x)=\frac{n!}{(n-i)!(n-1)!}\sum_{j=0}^{n-i}{\left(-1\right)}^{j}\left(\begin{array}{c} n-i\\ j \end{array}\right)f(x)F^{i+j-1}(x).$$

The series representation of the pdf in Equation (5) is given by;

(27) $$\begin{array}{cc} f(x)= & \;\alpha \beta (1-e^{-\lambda })e^{-\beta (1-e^{-\lambda })x}\sum_{k=0}^{\infty }\left(\begin{array}{c} \alpha \\ k \end{array}\right){\left(-1\right)}^{k}e^{-k\beta (1-e^{-\lambda })x}\\ & \times \sum_{\ell =0}^{\infty }{\left(-1\right)}^{\ell }\left(\begin{array}{c} 1+\ell -1\\ \ell \end{array}\right){\left(-1\right)}^{\ell }e^{-\ell \beta (1-e^{-\lambda })x} \end{array}$$

which simplifies to

(28) $$f(x)=\alpha \beta (1-e^{-\lambda })\sum_{k=0}^{\infty }\sum_{\ell =0}^{\infty }{\left(-1\right)}^{k+2\ell }\left(\begin{array}{c} \alpha \\ k \end{array}\right)e^{-\beta (1-e^{-\lambda })[k+\ell ]x},$$

the (i+j−1)*th* power of the *cdf* in Equation (4) in series representation is given by;

(29) $$\begin{array}{ccc} F^{i+j-1}(x) & = & {\left(1-e^{-\beta (1-e^{-\lambda })x}\right)}^{\alpha (i+j-1)}\\ & = & \sum_{m=0}^{\infty }\left(\begin{array}{c} \alpha (i+j-1)\\ m \end{array}\right){\left(-1\right)}^{m}e^{-m\beta (1-e^{-\lambda })x}. \end{array}$$

Substituting Equation (28) and (29) into Equation (26) we have the density of the *ith* order statistics in Equation (30)

(30) $$f_{i,n}(x)=\frac{n!\alpha \beta (1-e^{-\lambda })}{(n-i)!(n-1)!}\varpi_{j,k,\ell ,m}e^{-\beta (1-e^{-\lambda })[k+\ell +m]x},$$

(31) $$f_{1,n}(x)=n\alpha \beta (1-e^{-\lambda })\varpi_{j,k,\ell ,m}^{\prime }e^{-\beta (1-e^{-\lambda })[k+\ell +m]x},$$

(32) $$f_{n,n}(x)=n\alpha \beta (1-e^{-\lambda })\varpi_{k,\ell ,m}e^{-\beta (1-e^{-\lambda })[k+\ell +m]x}$$

while Equation (31) and Equation (32) correspond to the density of the smallest and largest order statistics respectively, where

$$\begin{array}{ccc} \varpi_{j,k,\ell ,m} & = & \sum_{j=0}^{n-i}\sum_{k=0}^{\infty }\sum_{\ell =0}^{\infty }\sum_{m=0}^{\infty }{\left(-1\right)}^{j+k+2\ell +m}\left(\begin{array}{c} n-i\\ j \end{array}\right)\left(\begin{array}{c} \alpha \\ k \end{array}\right)\left(\begin{array}{c} \alpha (i+j-1)\\ m \end{array}\right),\\ \varpi_{j,k,\ell ,m}^{\prime } & = & \sum_{j=0}^{n-i}\sum_{k=0}^{\infty }\sum_{\ell =0}^{\infty }\sum_{m=0}^{\infty }{\left(-1\right)}^{j+k+2\ell +m}\frac{\alpha !(\alpha j)!}{j!k!m!(n-1-j)!(\alpha -k)!(\alpha j-m)!},\;\text{and}\\ \varpi_{k,\ell ,m} & = & \sum_{k=0}^{\infty }\sum_{\ell =0}^{\infty }\sum_{m=0}^{\infty }{\left(-1\right)}^{k+2\ell +m}\frac{\alpha !\alpha (n-1)!}{k!m!(\alpha -k)!(\alpha (n-1)-m)!}. \end{array}$$

Theorem 2.7 *The pth crude moment of* $X_{i,n}\;(E(X_{i,n}^{p}))$ *is given by;*

$$E(X_{i,n}^{p})=\frac{\varpi_{j,k,\ell ,m,n}\Gamma (p+1)}{{\left[\beta (1-e^{-\lambda })(k+\ell +m)\right]}^{p+1}}.$$

Proof Using Equation (30) we have

(33) $$E(X_{i,n}^{p})=\varpi_{j,k,\ell ,m,n}\int_{0}^{\infty }x^{p}e^{-\beta (1-e^{-\lambda })[k+\ell +m]x}dx,$$

substituting $y=\beta (1-e^{-\lambda })[k+\ell +m]x$ into Equation (33) we have

$$\begin{array}{ccc} E(X_{i,n}^{p}) & = & \frac{\varpi_{j,k,\ell ,m,n}}{{\left[\beta (1-e^{-\lambda })(k+\ell +m)\right]}^{p+1}}\int_{0}^{\infty }y^{p}e^{-y}dy\\ & = & \frac{\varpi_{j,k,\ell ,m,n}\Gamma (p+1)}{{\left[\beta (1-e^{-\lambda })(k+\ell +m)\right]}^{p+1}}, \end{array}$$

where

$$\begin{array}{c} \varpi_{j,k,\ell ,m,n}\\ \;=\frac{n!\alpha \beta (1-e^{-\lambda })}{(n-i)!(n-1)!}\sum_{j=0}^{n-i}\sum_{k,\ell ,m=0}^{\infty }{\left(-1\right)}^{j+k+2\ell +m}\left(\begin{array}{c} n-i\\ j \end{array}\right)\left(\begin{array}{c} \alpha \\ k \end{array}\right)\left(\begin{array}{c} \alpha (i+j-1)\\ m \end{array}\right). \end{array}$$

 □

## 3 Calculation

In this section, we shall discuss the maximum likelihood estimation procedure for estimating the parameters of the **EETE** distribution.

Suppose the random sample $x_{1},x_{2},x_{3},\ldots ,x_{n}$ of size *n* is drawn from the **EETE** distribution with *pdf* f(x) in Equation (5) then the maximum likelihood estimates (mle) of its parameters could be obtained as follows:

The likelihood (L) equation is given by;

(34) $$\begin{array}{ccc} L & = & \prod_{i=1}^{n}\alpha \beta (1-e^{-\lambda })e^{-\beta (1-e^{-\lambda })x_{i}}{\left(1-e^{-\beta (1-e^{-\lambda })x_{i}}\right)}^{\alpha -1}\\ & = & {\left[\alpha \beta (1-e^{-\lambda })\right]}^{n}e^{-\beta (1-e^{-\lambda })\sum_{i=1}^{n}x_{i}}\prod_{i=1}^{n}{\left(1-e^{-\beta (1-e^{-\lambda })x_{i}}\right)}^{\alpha -1} \end{array}$$

and the log-likelihood function is given by;

(35) $$\begin{array}{ccc} \ell & = & n\log[\alpha \beta (1-e^{-\lambda })]\\ & & -\beta (1-e^{-\lambda })\sum_{i=1}^{n}x_{i}+(\alpha -1)\sum_{i=1}^{n}\log\left(1-e^{-\beta (1-e^{-\lambda })x_{i}}\right). \end{array}$$

Taking the partial derivatives of Equation (35) with respect to α,β and *λ* and setting resulting equations to zero, we obtain a system of three equations in three unknowns given as follows;

(36) $$\frac{\partial \ell }{\partial \alpha }=\frac{n}{\alpha }+\sum_{i=1}^{n}\log(1-e^{-\beta (1-e^{-\lambda })x_{i}})=0,$$

(37) $$\frac{\partial \ell }{\partial \beta }=\frac{n}{\beta }-(1-e^{-\lambda })\sum_{i=1}^{n}x_{i}+(\alpha -1)(1-e^{-\lambda })\sum_{i=1}^{n}\frac{x_{i}e^{-\beta (1-e^{-\lambda })x_{i}}}{1-e^{-\beta (1-e^{-\lambda })x_{i}}}=0,$$

(38) $$\frac{\partial \ell }{\partial \lambda }=\frac{ne^{-\lambda }}{1-e^{-\lambda }}-\beta e^{-\lambda }\sum_{i=1}^{n}x_{i}+(\alpha -1)\beta e^{-\lambda }\sum_{i=1}^{n}\frac{x_{i}e^{-\beta (1-e^{-\lambda })x_{i}}}{1-e^{-\beta (1-e^{-\lambda })x_{i}}}=0.$$

At this point, it is important to highlight that the analytical solutions of the system of nonlinear equations in Equations (36), (37) and (38) are unknown. Hence, the estimates $\overset{ˆ}{\alpha },\;\overset{ˆ}{\beta }$ and $\overset{ˆ}{\lambda }$ can only be obtained by solving the system by some numerical methods.

Let $\Omega ={\left(\overset{ˆ}{\alpha },\;\overset{ˆ}{\beta },\;\overset{ˆ}{\lambda }\right)}^{\prime }$. Under some standard regularity conditions, $\sqrt{n}(\overset{ˆ}{\Omega }-\Omega )$ is asymptotically multivariate normal distributed $N_{3}(0,J_{n}^{-1}(\Omega ))$, where $J_{n}(\Omega )$ is the expected information matrix defined by $E(\partial^{2}\ell (\Omega )/\partial \Omega \partial \Omega^{\prime })$. The asymptotic behavior of the expected information matrix can be approximated by the observed information matrix, denoted by $I_{n}(\overset{ˆ}{\Omega })$. Generally speaking, the diagonal elements of $I_{n}^{-1}(\overset{ˆ}{\Omega })$ give the variance of $\left(\overset{ˆ}{\Omega }\right)$ while the off-diagonal elements are the covariances. The observed information matrix of the **EETE** distribution is given by;

$$I_{n}(\overset{ˆ}{\Omega })=\left(\begin{array}{ccc} \frac{\partial^{2}\overset{ˆ}{\ell }(\Omega )}{\partial \alpha^{2}} & \frac{\partial^{2}\overset{ˆ}{\ell }(\Omega )}{\partial \alpha \partial \beta } & \frac{\partial^{2}\overset{ˆ}{\ell }(\Omega )}{\partial \alpha \partial \lambda }\\ \frac{\partial^{2}\overset{ˆ}{\ell }(\Omega )}{\partial \beta \partial \alpha } & \frac{\partial^{2}\overset{ˆ}{\ell }(\Omega )}{\partial \beta^{2}} & \frac{\partial^{2}\overset{ˆ}{\ell }(\Omega )}{\partial \beta \partial \lambda }\\ \frac{\partial^{2}\overset{ˆ}{\ell }(\Omega )}{\partial \lambda \partial \alpha } & \frac{\partial^{2}\overset{ˆ}{\ell }(\Omega )}{\partial \lambda \partial \beta } & \frac{\partial^{2}\overset{ˆ}{\ell }(\Omega )}{\partial \lambda^{2}} \end{array}\right).$$

The corresponding entries of the observed information matrix $I_{n}(\overset{ˆ}{\Omega })$ are:

$$\begin{array}{ccc} \frac{\partial^{2}\ell (\Omega )}{\partial \alpha^{2}} & = & -\frac{n}{\alpha^{2}},\\ \frac{\partial^{2}\ell (\Omega )}{\partial \beta^{2}} & = & -\frac{n}{\beta^{2}}-(\alpha -1){\left(1-e^{-\lambda }\right)}^{2}\sum_{i=1}^{n}\left[\frac{x_{i}^{2}e^{-2\beta (1-e^{-\lambda })x_{i}}}{\left(1-e^{-\beta (1-e^{-\lambda })x_{i}}\right)}+\frac{x_{i}^{2}e^{-\beta (1-e^{-\lambda })x_{i}}}{1-e^{-\beta (1-e^{-\lambda })x_{i}}}\right],\\ \frac{\partial^{2}\ell (\Omega )}{\partial \lambda^{2}} & = & \frac{e^{-\lambda }}{{\left(e^{\lambda }-1\right)}^{2}}[\beta {\left(e^{\lambda }-1\right)}^{2}\sum_{i=1}^{n}x_{i}-e^{\lambda }[ne^{\lambda }-(\alpha -1){\left(e^{\lambda }-1\right)}^{2}\\ & & \times \sum_{i=1}^{n}\left(\frac{\beta x_{i}(-1-\beta x_{i}e^{-\lambda })e^{-\lambda -\beta (1-e^{-\lambda })}}{1-e^{-\beta (1-e^{-\lambda })x_{i}}}-\frac{\beta x_{i}^{2}e^{-2\lambda -2\beta (1-e^{-\lambda })x_{i}}}{{\left(1-e^{-\beta (1-e^{-\lambda })x_{i}}\right)}^{2}}\right)]],\\ \frac{\partial^{2}\ell (\Omega )}{\partial \alpha \partial \beta } & = & (1-e^{-\lambda })\sum_{i=1}^{n}\frac{x_{i}e^{-\beta (1-e^{-\lambda })x_{i}}}{1-e^{-\beta (1-e^{-\lambda })x_{i}}},\\ \frac{\partial^{2}\ell (\Omega )}{\partial \alpha \partial \lambda } & = & \beta \sum_{i=1}^{n}\frac{x_{i}e^{-\lambda -\beta (1-e^{-\lambda })x_{i}}}{1-e^{-\beta (1-e^{-\lambda })x_{i}}}, \end{array}$$

and

$$\begin{array}{ccc} \frac{\partial^{2}\ell (\Omega )}{\partial \beta \partial \lambda } & = & -e^{-\lambda }\sum_{i=1}^{n}x_{i}+(\alpha -1)\sum_{i=1}^{n}[\frac{x_{i}e^{-\lambda -\beta (1-e^{-\lambda })x_{i}}}{1-e^{-\beta (1-e^{-\lambda })x_{i}}}\\ & & -\frac{\beta (1-e^{-\lambda })x_{i}^{2}e^{-2\beta (1-e^{-\lambda })x_{i}}}{{\left(1-e^{-\beta (1-e^{-\lambda })x_{i}}\right)}^{2}}-\frac{\beta (1-e^{-\lambda }x_{i}e^{-\beta (1-e^{-\lambda })x_{i}})}{1-e^{-\beta (1-e^{-\lambda })x_{i}}}]. \end{array}$$

Given that $\sqrt{n}(\overset{ˆ}{\Omega }-\Omega )∼N_{3}(0,I_{n}^{-1}(\overset{ˆ}{\Omega }))$ statistical inference for functions of **Ω** can now be performed. For example, the approximate 100(1−ϵ)% two-sided confidence interval of the model parameters $\overset{ˆ}{\Omega }$ could be calculated as:

$$\overset{ˆ}{\Omega }\pm Z_{\frac{\epsilon }{2}}\sqrt{I_{\frac{\partial^{2}\ell (\Omega )}{\partial \Omega^{\prime }}}^{-1}(\cdot )},$$

where $I_{\frac{\partial^{2}\ell (\Omega )}{\partial \Omega^{\prime }}}^{-1}(\cdot )$ are the diagonal entries of the observed information matrix, and $Z_{\frac{\epsilon }{2}}$ is the upper ϵ/2th percentile of the standard normal distribution.

## 4 Results

In this section, we shall investigate the stability of the *mle* estimates of the parameters of the **EETE** distribution with different sample size *(n)* through a Monte-Carlo study, followed by a real-life example of possible application of the new distribution.

The simulation procedure as outlined below was performed in *R (Statistical software)*:

1. simulate a random sample of size *n* from the **EETE** distribution with parameters α=5, β=5 and λ=5 using the inversion of the *cdf* method with Equation (11).
2. set initial values for the parameters ($\alpha_{0},\;\beta_{0},\;\lambda_{0}$). Generally, no matter the choice of the initial values, the *mle* converges to the true parameter value, although, the rate of convergence may be affected. In this case, initial values close to the true values gives a quicker convergence, so we have always chosen initial parameter values of 4.5 for each parameter.
3. compute the *mle* of the parameters of the **EETE** distribution.
4. repeat steps 1–3 *5000* (*N*) times.
5. compute the mean, standard deviation (standard error), bias and mean square error *(MSE)* of the *5000* estimates of each parameter (α,β and *λ*).
6. repeat steps 1–5 with different sample sizes *(n* = *10, 20, 30, 40, 50,...,200).*

Here the *mle* estimates are computed as $\overset{¯}{\overset{ˆ}{\Omega }}=1/N\sum_{i=1}^{n}{\overset{ˆ}{\Omega }}_{i}$, standard error as $SE_{\overset{¯}{\overset{ˆ}{\Omega }}}=\sqrt{\sum_{i=1}^{N}{\left({\overset{ˆ}{\Omega }}_{i}-\overset{¯}{\overset{ˆ}{\Omega }}\right)}^{2}/(N-1)}$, bias as $bias={\overset{¯}{\overset{ˆ}{\Omega }}}_{i}-\Omega ;\;i=1,\ldots ,n$ and *MSE* as $MSE_{\overset{¯}{\overset{ˆ}{\Omega }}}=1/N\sum_{i=1}^{N}{\left({\overset{ˆ}{\Omega }}_{i}-\Omega \right)}^{2}$. Results from the Monte-Carlo simulation study are tabulated in Table 3, Table 4.

**Table 3:** Simulation results of the estimates and standard errors of the **EETE** distribution parameters for different sample sizes.

*n*	$\overset{¯}{\overset{ˆ}{\alpha }}$	$\overset{¯}{\overset{ˆ}{\beta }}$	$\overset{¯}{\overset{ˆ}{\lambda }}$	$SE_{\overset{¯}{\overset{ˆ}{\alpha }}}$	$SE_{\overset{¯}{\overset{ˆ}{\beta }}}$	$SE_{\overset{¯}{\overset{ˆ}{\lambda }}}$
10	11.706811	6.011519	4.987554	47.4959754	2.4326054	0.23557895
20	6.666512	5.450365	5.010185	4.5580701	1.2201133	0.04999422
30	5.932643	5.252735	5.006534	2.5613821	0.9533649	0.03057048
40	5.659878	5.195467	5.005612	1.9212756	0.8020279	0.02612018
50	5.552010	5.173986	5.005199	1.6241554	0.7098957	0.02349393
60	5.454993	5.153036	5.004536	1.4178006	0.6409210	0.02118106
70	5.378195	5.119426	5.003411	1.3050481	0.6012803	0.01989303
80	5.306566	5.107778	5.003089	1.1685310	0.5474992	0.01823719
90	5.276825	5.096462	5.002707	1.0914899	0.5144220	0.01721715
100	5.262917	5.085633	5.002375	1.0126719	0.4861546	0.01630660
110	5.232479	5.070903	5.001942	0.9742818	0.4636417	0.01549881
120	5.200665	5.067221	5.001915	0.9121694	0.4407050	0.01475293
130	5.195405	5.072106	5.002173	0.8919152	0.4361730	0.01463290
140	5.192058	5.066804	5.002017	0.8406262	0.4131714	0.01389369
150	5.156469	5.051550	5.001533	0.7921979	0.3935480	0.01323162
160	5.145878	5.045873	5.001349	0.7789221	0.3848671	0.01293368
170	5.144871	5.049913	5.001479	0.7482957	0.3676211	0.01239179
180	5.135238	5.046527	5.001376	0.7298677	0.3637811	0.01226390
190	5.120252	5.040198	5.001175	0.6985037	0.3479368	0.01175635
200	5.120010	5.042599	5.001258	0.6750239	0.3430995	0.01162621

**Table 4:** Simulation results of the bias and *MSE* of the **EETE** distribution parameters for different sample sizes.

*n*	$bias_{\overset{¯}{\overset{ˆ}{\alpha }}}$	$bias_{\overset{¯}{\overset{ˆ}{\beta }}}$	$bias_{\overset{¯}{\overset{ˆ}{\lambda }}}$	$MSE_{\overset{¯}{\overset{ˆ}{\alpha }}}$	$MSE_{\overset{¯}{\overset{ˆ}{\beta }}}$	$MSE_{\overset{¯}{\overset{ˆ}{\lambda }}}$
10	6.7068113	1.01151911	-0.012445545	2300.3978275	6.9395564	0.0556412341
20	1.6665115	0.45036465	0.010185459	23.5491085	1.6912071	0.0026026658
30	0.9326432	0.25273473	0.006534144	7.4291896	0.9725978	0.0009770622
40	0.6598776	0.19546703	0.005612190	4.1260000	0.6813274	0.0007136241
50	0.5520100	0.17398592	0.005199447	2.9420684	0.5341222	0.0005788886
60	0.4549926	0.15303616	0.004535626	2.2167749	0.4341177	0.0004691195
70	0.3781953	0.11942595	0.003410988	1.8458417	0.3757283	0.0004072884
80	0.3065664	0.10777820	0.003089203	1.4591746	0.3113116	0.0003420718
90	0.2768246	0.09646165	0.002707322	1.2677439	0.2738819	0.0003037005
100	0.2629171	0.08563286	0.002375256	1.0944246	0.2436320	0.0002714940
110	0.2324794	0.07090338	0.001941729	1.0030819	0.2199479	0.0002439355
120	0.2006647	0.06722145	0.001915141	0.8721529	0.1987007	0.0002212733
130	0.1954046	0.07210626	0.002172900	0.8335366	0.1954082	0.0002188003
140	0.1920581	0.06680399	0.002016622	0.7433974	0.1751392	0.0001970628
150	0.1564691	0.05154993	0.001532899	0.6519346	0.1575065	0.0001773905
160	0.1458780	0.04587345	0.001348894	0.6278787	0.1501975	0.0001690660
170	0.1448705	0.04991321	0.001479039	0.5808220	0.1376095	0.0001557133
180	0.1352379	0.04652673	0.001375921	0.5508897	0.1344750	0.0001522664
190	0.1202524	0.04019830	0.001174785	0.5022704	0.1226517	0.0001395643
200	0.1200102	0.04259927	0.001258358	0.4699686	0.1195084	0.0001367253

Here, we illustrate the applicability of the **EETE** distribution with a real data set. The goodness of fit of the new lifetime distribution would be assessed by means of comparing its fitting performance with those of

• **ETE** distribution; see Equation (2),
• Exponentiated Lomax distribution
  $$f(x)=\alpha sr{\left(1+rx\right)}^{-(s+1)}{\left[1-{\left(1+rx\right)}^{-s}\right]}^{\alpha -1},\;x\;,\alpha ,\;s,\;r>0,$$
• Exponentiated Burr XII distribution
  $$f(x)=\alpha kcx^{c-1}{\left(1+x^{c}\right)}^{-k-1}{\left[1-{\left(1+x^{c}\right)}^{-k}\right]}^{\alpha -1},\;x\;,\alpha ,\;c,\;k>0,$$
• Exponentiated Fréchet distribution
  $$f(x)=\frac{\alpha \beta }{s}{\left(\frac{x}{s}\right)}^{-\beta -1}e^{-\alpha {\left(\frac{x}{s}\right)}^{-\beta }},\;x\;,\alpha ,\;\beta ,\;s>0\;\;\text{and}$$
• Transmuted Erlang-Truncated Exponential (**TETE**) distribution
  $$\begin{array}{cc} f(x)= & \;\beta \left(1-e^{-\lambda }\right)e^{-\beta (1-e^{-\lambda })x}\\ & \times \left(1+\alpha -2\alpha e^{-\beta (1-e^{-\lambda })x}\right),\;|\alpha |\leq 1,\;\beta ,\lambda >0\;,\text{ and }0\leq x<\infty . \end{array}$$

Comparison of the fitted models would be based on the following goodness of fit measures: the Akaike information criterion (AIC), Akaike [20], the Bayesian information criterion (BIC), Schwarz [21] and the generalized Cramér–von Mises $W^{⋆}$ statistics; due to Chen and Balakrishnan[22] and Pakyari and Balakrishnan [23]. The smaller the criterion statistics the better the model. A graphical technique via a Probability–Probability (P–P) plot is also considered to visually compare the empirical distribution against the theoretical distribution.

The real data set in Table 5 shows the annual maximum daily precipitation in millimeter that was recorded in Basan, Korea, from 1904 to 2011. The data have been analyzed by Jeong et al. [24] and were recently reported in Mansoor et al. [25]. Results obtained from fitting the distributions are presented in Table 6.

**Table 5:** Rainfall data.

24.8	140.9	54.1	153.5	47.9	165.5	68.5	153.1	254.7	175.3	87.6	150.6
147.9	354.7	128.5	150.4	119.2	69.7	185.1	153.4	121.7	99.3	126.9	150.1
149.1	143	125.2	97.2	179.3	125.8	101	89.8	54.6	283.9	94.3	165.4
48.3	69.2	147.1	114.2	159.4	114.9	58.5	76.6	20.7	107.1	244.5	126
122.2	219.9	153.2	145.3	101.9	135.3	103.1	74.7	174	126	144.9	226.3
96.2	149.3	122.3	164.8	188.6	273.2	61.2	84.3	130.5	96.2	155.8	194.6
92	131	137	106.8	131.6	268.2	124.5	147.8	294.6	101.6	103.1	247.5
140.2	153.3	91.8	79.4	149.2	168.6	127.7	332.8	261.6	122.9	273.4	178
177	108.5	115	241	76	127.5	190	259.5	301.5

**Table 6:** Results from modelling the rainfall data.

Models	Estimates	SE	$-\overset{ˆ}{\ell }$	AIC	**BIC**	$W^{⋆}$
**EETE**			582.3911	1170.782	1178.744	0.426215
$\overset{ˆ}{\alpha }$	6.27482	1.1480640				
$\overset{ˆ}{\beta }$	0.08602	0.2808922				
$\overset{ˆ}{\lambda }$	0.22181	0.8111474				

**ETE**			627.2663	1258.533	1263.841	104.360900
$\overset{ˆ}{\beta }$	0.01535	0.0165117				
$\overset{ˆ}{\lambda }$	0.59875	0.8855350				

**ExpLomax**			587.0722	1180.144	1188.106	4.104561
$\overset{ˆ}{\alpha }$	10.09662	2.0641755				
$\overset{ˆ}{r}$	0.00507	0.0007164				
$\overset{ˆ}{s}$	5.45858	0.6292469				

**ExpBurr XII**			612.2200	1230.440	1238.402	2.417171
$\overset{ˆ}{\alpha }$	591.16829	181.9098298				
$\overset{ˆ}{k}$	0.93487	0.4196369				
$\overset{ˆ}{c}$	1.47831	0.6605398				

$ExpFr\overset{´}{e}chet$			608.5969	1223.194	1231.156	1.225102
$\overset{ˆ}{\alpha }$	14.21029	227.2744212				
$\overset{ˆ}{\beta }$	1.64995	0.1031434				
$\overset{ˆ}{s}$	19.99516	193.8275346				

**TETE**			600.5034	1207.007	1214.969	2121.515000
$\overset{ˆ}{\alpha }$	0.999999	–				
$\overset{ˆ}{\beta }$	0.046098	–				
$\overset{ˆ}{\lambda }$	0.248734	–				

The variance–covariance matrix of the **EETE** distribution under the fitted rainfall data is given by;

$$I_{n}^{-1}(\overset{ˆ}{\Omega })=\left(\begin{array}{ccc} 1.318050826 & 0.001855195 & 0.0159270\\ 0.001855195 & 0.078900440 & -0.2277618\\ 0.015927002 & -0.227761824 & 0.6579601 \end{array}\right).$$

## 5 Discussion

**Figure 3:**
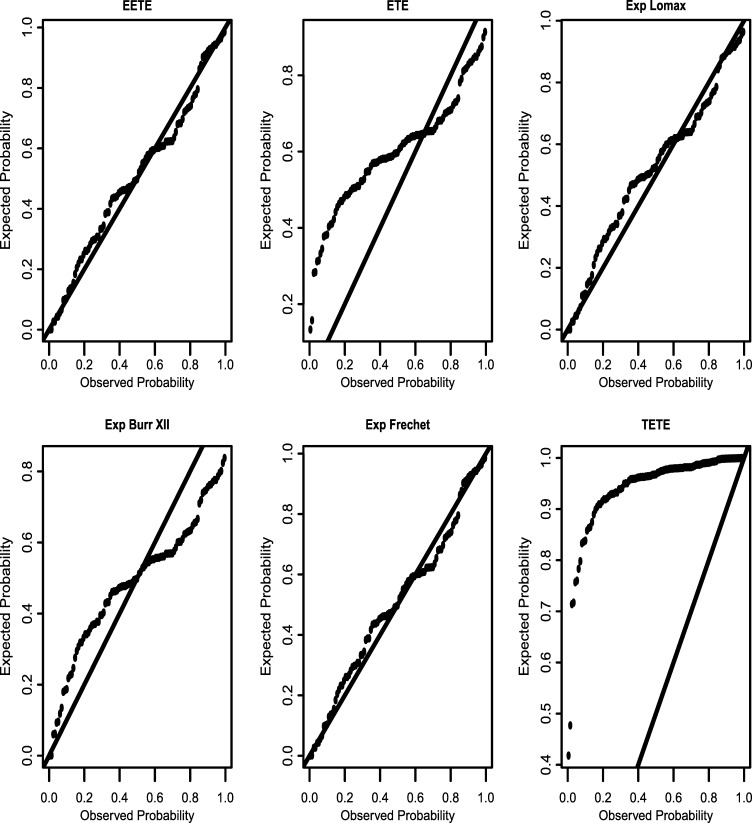
P–P plots of the fitted distributions in Table 6.

The **EETE** is suitable for analyzing decreasing and unimodal data sets and the failure rate function could be increasing, decreasing, or constant depending on the shape parameter (*α*). This characteristics makes it more sophisticated for modelling data sets from various complex lifetime phenomena especially those with early time failure characteristics. The Monte-Carlo simulation results in Table 3, Table 4 show a reasonable consistency of the *mle* estimates. The results in Table 6 show that the **EETE** distribution with smaller minimized log-likelihood, AIC, BIC and $W^{⋆}$ statistics provides a better fit to the data set than the competing distributions. Also, it is clear from the plots in Figure 3 that the *cdf* of the **EETE** distribution fits the empirical *cdf* better than the **ETE**, Exponentiated Lomax, Exponentiated Burr XII, Exponentiated Fréchet and **TETE** distributions.

## 6 Conclusion

This paper introduces a new lifetime distribution – the Extended Erlang-Truncated Exponential **EETE** distribution. The new distribution generalizes the Erlang-Truncated Exponential **ETE** distribution and has the **ETE** distribution as a special case. We have given explicit mathematical expressions for some of its basic statistical properties such as the probability density function, cumulative density function, *kth* raw moment, *rth* incomplete moment, *kth* central moment, mean, variance, coefficient of variation, skewness, kurtosis, moment generating function, *qth* quantile function, the *ith* order statistics, and the Rényi's entropy measure. Also, some of its reliability characteristics like the reliability function and the failure rate function are provided. Estimation of the model parameters was approached through the method of maximum likelihood estimation and a Monte-Carlo simulation was performed to verify the stability of the *mle* estimates of the model parameters . The applicability and goodness of fit of the **EETE** distribution was illustrated with a real data set and the results obtained show that the **EETE** distribution is a better candidate for the data than the other competing distributions.

## Declarations

### Author contribution statement

Idika Okorie: Conceived and designed the experiments; Performed the experiments; Analyzed and interpreted the data; Contributed reagents, materials, analysis tools or data; Wrote the paper.

Anthony Akpanta, Johnson Ohakwe, David Chikezie: Performed the experiments; Analyzed and interpreted the data; Contributed reagents, materials, analysis tools or data; Wrote the paper.

### Funding statement

This research did not receive any specific grant from funding agencies in the public, commercial, or not-for-profit sectors.

### Competing interest statement

The authors declare no conflict of interest.

### Additional information

No additional information is available for this paper.
